# Investigating Social Competence in a Pilot Randomized Clinical Trial of a Theatre-Based Intervention Enhanced for Adults with Autism Spectrum Disorder

**DOI:** 10.1007/s10803-023-06214-0

**Published:** 2023-12-18

**Authors:** Blythe A. Corbett, Alexandra P. Key, Mark E. Klemencic, Rachael A. Muscatello, Dorita Jones, Jennifer Pilkington, Christina Burroughs, Simon Vandekar

**Affiliations:** 1 Department of Psychiatry and Behavioral Sciences, Vanderbilt University Medical Center, Nashville, TN USA; 2 Vanderbilt University Medical Center, Vanderbilt Kennedy Center, Nashville, TN USA; 3 Department of Psychology, Vanderbilt University, Nashville, TN USA; 4 Department of Hearing and Speech Sciences, Vanderbilt University Medical Center, Nashville, TN USA; 5 Department of Biostatistics, Vanderbilt University Medical Center, Nashville, TN USA

**Keywords:** Autism, Social competence, Event related potentials, Social salience

## Abstract

Autism spectrum disorder (ASD) is characterized by challenges in social competence that persist in adulthood, yet few treatment options exist. A pilot randomized clinical trial (RCT) of a peer-mediated, theatre-based intervention with established efficacy in youth with ASD was examined in autistic adults. The final sample consisted of forty-seven 18-to-40-year-old participants randomized to the experimental (EXP *N* = 23) or waitlist control (WLC *N* = 24) condition. A multimodal, social interdependent model was employed to examine social competence changes in brain (incidental face memory (IFM) using event-related potentials), cognition (Wechsler Memory Scale-III), behavior (Contextual Assessment of Social Skills) and function (Social Responsiveness Scale (SRS); Adaptive Behavior Assessment Scale (ABAS) Social Composite). Using analysis of covariance in which pretest was controlled in the model, posttest between-group differences were observed on IFM (*p* = 0.016, η^2^ = 0.139, d = 0.79) and several social and adaptive functional (SRS, ABAS) outcomes in social communication and interaction (SCI) (*p* = 0.019, η^2^ = 0.121, d = -00.45), communication (*p* = 0.044 η^2^ = 0.09, d = -00.31), and motivation (*p* = 0.001, η^2^ = 0.229, d = -0.79) domains. At two-month follow-up, gains in social motivation remained (*p* = 0.041, η^2^ = 0.100, d = -0.77). The results offer preliminary support for a unique theatre-based social skills intervention for autistic adults who have few treatment options to enhance social competence. The trial was pre-registered with ClinicalTrials.gov (Identifier: NCT04349644).

## Introduction

Autism spectrum disorder (ASD)^1^ is a neurodevelopmental condition characterized by core challenges in social competence (APA, 2013), involving neural, cognitive, behavioral, and functional components (Kennedy & Adolphs, 2012). Currently, the Centers for Disease Control estimates that 1:36 children are diagnosed with ASD (Maenner et al., 2023) and each year approximately 50,000 individuals with ASD turn 18 years old to enter adulthood (Shattuck et al., 2014). If untreated, difficulties in social competence are often intractable, persist into adulthood (Seltzer et al., 2004) and may worsen with age (Beadle-Brown et al., 2002). The worsening of challenges has been corroborated by autistic adults who report their social problems often peak in middle adulthood (Lever & Geurts, 2018). Meanwhile, social demands expand during the adult years intensifying the need for ongoing treatment. There is evidence that after secondary school, adults with ASD become increasingly more isolated from social activities in the community (Myers et al., 2015; Taylor et al., 2017; Umagami et al., 2022). Despite the growing need of this expanding population, after 18 years of age, services for persons with ASD decline significantly and are inadequate to meet their needs (Buescher et al., 2014; Dudley et al., 2018; Howlin & Magiati, 2017; Shattuck et al., 2011; Taylor & Henninger, 2015; Turcotte et al., 2016). Unfortunately, per-person spending for employment support, day care and transportation is highest for adults with ASD (Leigh et al., 2016).

While there has been some progress in recent years, effective psychosocial interventions for adults are scarce (Kandalaft et al., 2013; Koehne et al., 2016; Laugeson et al., 2012; Spain & Blainey, 2015; Taylor et al., 2012; White et al., 2015), with only a few randomized control trials (RCTs) (Ashman et al., 2017; Gantman et al., 2012; Oswald et al., 2018). Most social skill interventions for adults with ASD follow a similar format, incorporating didactics, small and large group discussions, practice exercises (e.g., role play), and other activities related to the didactic content (e.g., identifying social cues in an example). Studies are generally small, non-randomized or quasi-experimental (Ashman et al., 2017; Gantman et al., 2012; McVey et al., 2016; Oswald et al., 2018; Turner-Brown et al., 2008). Overall, results are promising—all report improvement or trends toward improvement on measures of social skills— but gains are limited (e.g., Ashman et al., 2017; Koehne et al., 2016; Oswald et al., 2018; Spain & Blainey, 2015). In addition, studies highlight the challenge of generalizability or clinical efficacy. For example, Ashman (2017) noted that, though social cognition improved per clinician-administered measures, improvement did not translate to contexts outside the study. Relatedly, McVey (2016) found significant improvement in social skill knowledge, self-report measures of empathy and social anxiety, and parent-reported social skills after PEERS®, a social skills treatment for adults. However, participants did not indicate any improvement in reported loneliness, suggesting difficulty with implementing their new skills for friendships. Turner-Brown (2008) used behavioral observation to look at multiple components of social communication yet found no significant post-intervention improvement. The lack of evidence for the generalizability of these interventions becomes even more problematic in the face of service gaps that are nearly ubiquitous among adults with ASD (Wong et al., 2015). Together, these studies portray a small body of research evaluating programs that seem to be effective in improving the specific skills targeted but have limited generalizability to the real-world, daily life of adults with ASD. Logical next steps would be to examine novel and ecologically valid interventions utilizing objective and multimodal measures.

The social interdependent model (Kennedy & Adolphs, 2012) posits that to understand social competence, four levels of analysis are warranted, extending from the social brain that underlies social cognition, which produces and modulates social behavior and ultimately establishes generalized social functioning. The social brain subserves the complex integration of social signals and is measured via imaging techniques such as Event-Related Potentials (ERP). Social cognition pertains to processing that is signaled by and directed toward other people and measured by examining the perception of key social signals, such as faces. Social behavior is generally measured through direct observation of the individual interacting with others within a given context (Kennedy & Adolphs, 2012). Finally, social functioning pertains to the integration of cognition, affect, and interactions reflecting the daily way an individual negotiates the social world measured by self-report of informants reporting adaptive skills in the daily environment (Constantino & Gruber, 2005; Harrison & Oakland, 2000). Social functioning is also thought to be comprised of social motivation, cognition, and interaction skills (Pallathra et al., 2018). Collectively, leading theories in ASD (Baron-Cohen, 1995; Chevallier et al., 2012; Osterling et al., 2002) generally support the social interdependent framework (Adolphs, 2001; Kennedy & Adolphs, 2012) such that ASD may be conceptualized as a disorder of social competence with measurable challenges regarding social cognition, behavior and functioning.

The *Social Motivation Theory* of autism speculates that early-onset challenges in social attention lead to a disruption in social learning experiences (Chevallier et al., 2012). Interventions that enhance social motivation via increasing the salience of or intrinsic interest in social stimuli should enhance performance in social functioning (Chevallier et al., 2012). Memory for faces is proposed as an index of social salience, a skill dependent on sufficient engagement with the stimuli and allocation of adequate processing resources (i.e., beyond initial stimulus detection and categorization). Therefore, face recognition and memory constitute a foundational building block for the development of age-appropriate social competence (Gauthier & Nelson, 2001; Nelson, 2001), facilitating social interactions, and creating social bonds. Remembering whether a face has been seen aids accessing information about past interactions, informs the selection of adaptive social behavior, and significantly improves the probability of social success (Corbett et al., 2014a; Hauck, 1998).

Research has shown that most individuals with ASD have particular difficulty remembering faces, especially following a delay (Griffin et al., 2021; Key & Corbett, 2014; Langdell, 1978; Osterling et al., 2002; Webb et al., 2010; Weigelt et al., 2012). Difficulty in face memory in people with ASD infer risk for development of social impairment (Marcus & Nelson, 2001). Behavioral and neuroimaging studies show that struggles with immediate and delayed recall of faces in persons with ASD persist into adulthood (O’Hearn et al., 2014; Williams et al., 2005). Difficulty with face identification in ASD is primarily observed on tasks with a memory component (Weigelt et al., 2012). For example, on the Wechsler Memory Scale-III (Wechsler, 2009), adults with ASD exhibited impaired memory for faces and everyday family scenes, but not for words or stories (Williams et al., 2005). In contrast to neurotypical same-age peers who continue to improve in face recognition, adults with ASD show increasing problems from adolescence to adulthood, presumably due to a developmental plateau (O’Hearn et al., 2014). The lack of improvement with age suggests unique and persistent structural and functional brain maturation patterns into adulthood in ASD, which can impact cognitive and social functioning (Floris et al., 2021; Hashem et al., 2020; Hyde et al., 2010; Johnson, 2017; Shafritz et al., 2008). As such, the lack of prototypical development widens the gap as adults with ASD fall further behind (Taylor & Seltzer, 2010). Finally, Lynn and colleagues (2018) showed age-related changes in adults with ASD relative to TD adults with respect to reduced connectivity between the Fusiform Face Area and subcortical and temporoparietal regions, and the amygdala. Thus, face memory challenges persist in adults with ASD and are influenced by task (delay) and developmental (age) factors.

Despite consistently noted difficulties, face memory has been shown to be modifiable by a theatre-based intervention called SENSE Theatre® (Corbett et al., 2011, 2014b, 2014c) and linked to improved functional outcomes of social competence (Corbett et al., 2011, 2014c, 2016). Using a randomized clinical trial (RCT), youth in the SENSE Theatre® experimental (EXP) group relative to the wait-listed control (WLC) group [AUTHORS BLINDED] demonstrated improvement in delayed memory for faces (MFD NEPSY; Korkman et al., 2007) (*d* = 0.98) and an event-related potential (ERP) Incidental Face Memory (IFM) task (*d* = 0.93). Face memory was strongly correlated with SRS Communication (index of social functioning) at posttest (r=-0.49, *p* = 0.01) and at follow-up (r=-0.47, *p* = 0.02) (Corbett et al., 2016). Recently, in a large multisite RCT of SENSE Theatre® compared to an active control condition (ACC), better IFM at posttest mediated increased social behavior at follow-up (Corbett et al., 2023). Taken together, it has been hypothesized that engaging in peer-mediated theatre activities increases attention to reinforcing social stimuli (supportive peers) such that participants are increasingly drawn to peers’ faces (increased salience) resulting in more generalized social attention to other people (Corbett et al., 2014c). These findings provide support for the predictability and strength of the interconnected social competence framework as well as the feasibility and utility of using face memory as a treatment target of social competence.

The goal of the current study was to address the critical unmet need for the development of efficacious psychosocial interventions for adults with ASD by utilizing an innovative intervention with emerging efficacy in children and adolescents (Corbett et al., 2011, 2014c, 2016). SENSE Theatre® combines established behavioral techniques alongside theatrical strategies delivered in a peer-mediated, community service model with strong evidence of significant improvement in social competence (i.e., face memory; (Corbett et al., 2011, 2014c, 2016)) as well as changes in stress (Corbett et al., 2014c) and anxiety (Corbett et al., 2016) in children and adolescents with ASD. While previous research has been rigorous and efficacious in youth, the objective of the study was to determine if SENSE Theatre® is effective in impacting salience of social information, which in turn results in improved functional outcomes for adults with ASD.

### Purpose

The purpose of the present study was to examine and extend the impact of SENSE Theatre® on adults with ASD using a randomized (EXP vs. WLC) design measuring social competence across the interdependent model involving neural, cognitive, behavioral and functional levels. Measurements were conducted at pretest, posttest and following a two-month follow-up period. Based on previous studies, the following Hypotheses (Hyp) were made. Hyp 1. Adults with ASD in the EXP group will demonstrate significantly greater posttest IFM index (primary dependent variable (DV)) than the WLC group. Hyp 2.a Adults in the EXP group will demonstrate significantly better posttest social cognition (i.e., Wechsler Memory Scale) and Hyp 2.b social behavior than adults in the WLC (i.e., Vocal Expression, Quality of Rapport). Hyp 3. Adults in the EXP group will demonstrate significantly more growth on functional social outcomes (Social Responsiveness Scale (SRS), Adaptive Behavior Assessment Scale (ABAS)). Hyp 4. The observed gains in social competence at posttest will be maintained at follow-up.

## Methods

The research was carried out in accordance with the Code of Ethics of the World Medical Association (Declaration of Helsinki). The Vanderbilt University Institutional Review Board approved the study. Informed written consent was obtained from adult participants and a Surrogate Rider consent was obtained for six legally conserved participants prior to inclusion in the study.

### Participants

Participants were recruited from a broad community sample via targeted outreach to medical and health-related services, clinics, research registries, regional autism/disability organizations, schools, and social media platforms. Inclusion criteria for the sample required a confirmed diagnosis of ASD based on DSM-5 criteria (APA, 2013) by a psychologist, pediatrician or psychiatrist with expertise in ASD, which was corroborated by the Autism Diagnostic Observation Schedule (ADOS-2) (Lord et al., 2012). Participants were required to have a full-scale IQ ≥ 70 (WASI; Wechsler, 2011). Adults with ASD with intellectual disability were excluded from the study because it is unclear if they will benefit from the intervention due to the demands on broad average cognitive and language ability. Moreover, previous research examining the efficacy of the treatment with youth has been conducted on individuals with IQ ≥ 70 (Corbett et al., 2016, 2023). Participants with current, frequent, and uncontrolled aggression toward other persons or property in the past six months were excluded based on phone screening and questions from the Adult Behavior Checklist (Achenbach, 2001) (e.g., “Physically attacks people”). The rationale for exclusion based on aggressive behavior was to ensure the safety of all participants, peers and staff.

Participants were assigned to either the EXP or WLC across 3 cohorts using a block randomization table created by the university statistics core independent from the research team. Following consent, participants received an ID number that corresponded to group assignment for that number. To conceal group allocation from assessors and coders, individuals naïve to treatment assignment conducted outcome assessments.

**Fig. 1:**
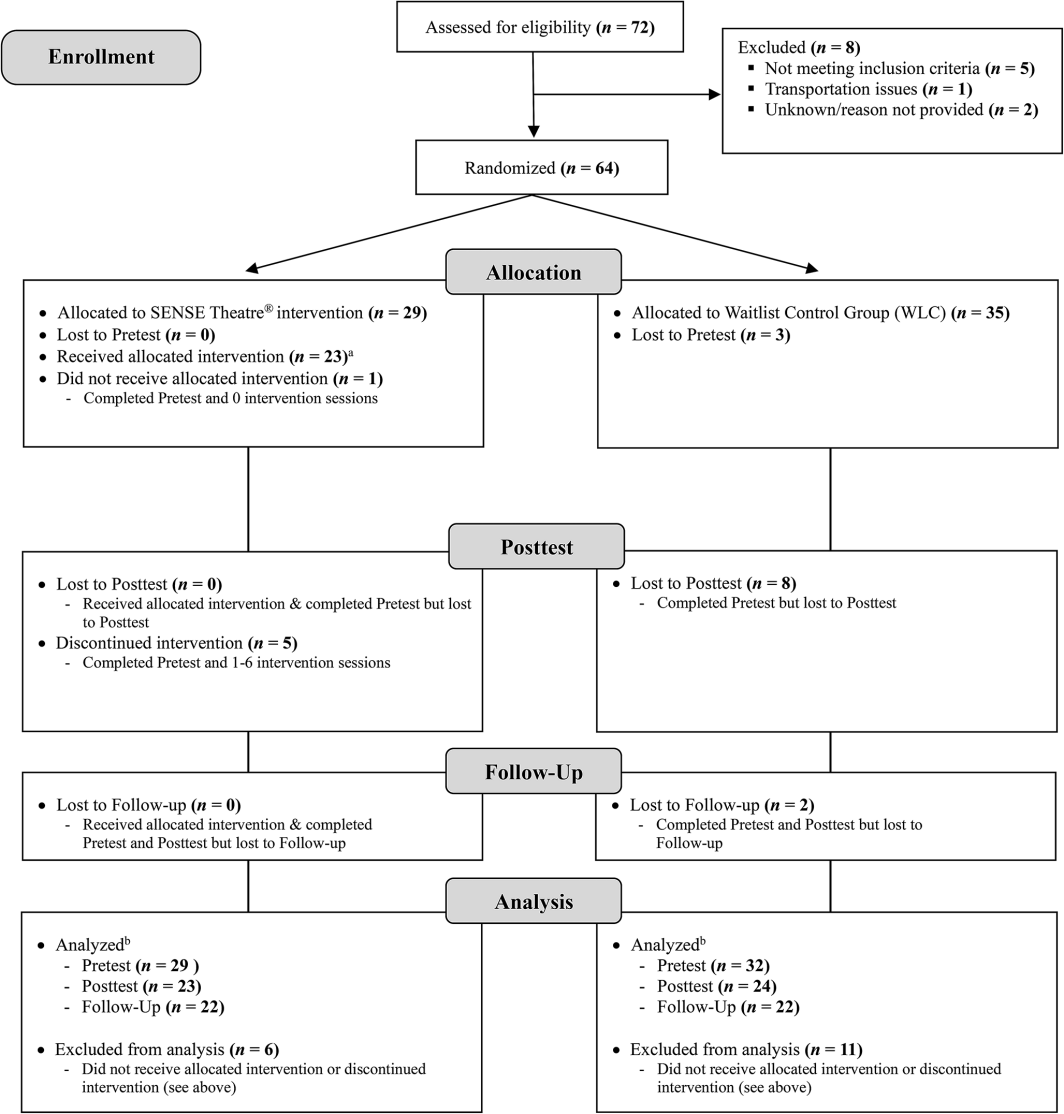
Consort Diagram. Note: CONSORT = Consolidated Standards of Reporting Trials; Footnotes: ^a^ Completed “per protocol” intervention (i.e., attended ≥ 7 intervention sessions), ^b^Data from subjects who completed “per protocol” intervention and at least one Primary or Secondary DV at Pretest, Posttest, or Follow-Up.

The eligible sample consisted of 64 participants between 18 and 40 years randomized to EXP (*N* = 29) or WLC (*N* = 35); however, analyses were conducted on a final sample of 47 participants who completed a per protocol number of ≥ 7 of 10 sessions (see CONSORT Diagram Fig. 1 for details). Power analyses using effect sizes from a previous recent RCT of the intervention (Corbett et al., 2016) indicated a target sample size of 40 subjects with 10% attrition is sufficient to obtain 80% power.

The demographic data are presented in Table 1. The mean age was 24.30 years across the groups. The sex distribution was nearly evenly distributed with 31 (48%) females and 33 (52%) males. The racial and ethnic characterization of the sample was comprised of 10.9% Black, 75.0% White, 1.6% Asian, and 12.5% multiracial. There were 4.7% Hispanic and 95.3% non-Hispanic participants.

**Table 1:** Demographic and pretest variables

Variable	EXP (*N* = 29)		WLC (*N* = 35)		Test Statistic	*p*
	*M*	*SD*	*M*	*SD*		
**Demographics**						
Age	24.48	4.92	24.30	5.97	t(62) = -0.001	0.999
WASI-II (FSIQ-4)	102.45	17.21	103.06	22.86	t(61) = -0.12	0.91
ADOS-2	7.34	1.86	7.51	1.62	t(62) = -0.39	0.698
SCQ-L	17.83	7.78	16.32	7.4	t(53) = 0.716	0.477
**Pretest Variables**						
ERP/IFM	0.2944	1.55727	0.106	1.91583	F(1,58) = 0.173	0.679
WMS-III (Faces II)	7.17	3.048	7.19	2.428	t(58) = -0.03	0.976
CASS						
Vocal Expressiveness	4.62	1.498	4.00	1.366	t(58) = 1.679	0.099
Quality of Rapport	4.83	1.441	4.59	1.132	t(59) = 0.708	0.482
SRS-2						
Total Score	67.04	11.107	67.00	9.829	t(57) = 0.013	0.990
Social Cognition	64.0	9.611	64.74	9.476	t(57) = -0.298	0.767
Social Communication	67.14	10.255	64.87	10.683	t(57) = 0.831	0.409
Social Motivation	64.39	12.05	67.74	11.43	t(57) = -1.095	0.278
Social Awareness	61.07	11.349	58.58	8.131	t(57) = 0.976	0.333
SCI	66.36	10.695	65.90	9.779	t(57) = 0.17	0.454
ABAS Social Composite	84.08	11.02	80.43	15.373	t(54) = 1.005	0.319

*Note.* EXP = Experimental Group; WLC = Waitlist Control Group; WASI-II = Wechsler Abbreviated Scale of Intelligence Second Edition; ADOS-2 = Autism Diagnostic Observation Schedule, Second Edition; SCQ-L = Social Communication Questionnaire, Lifetime Form; ERP/IFM = Event Related Potential/Incidental Face Memory; WMS-III (Faces II) = Wechsler Memory Scale, Third Edition—Faces II Subtest; CASS = Contextual Assessment of Social Skills; SRS-2 = Social Responsiveness Scale, Second Edition (SCI Subscale = Social Communication and Interaction); ABAS-3 = Adaptive Behavior Assessment System Third Edition

### Diagnostic Procedures

*Autism Diagnostic Observation Schedule* (ADOS-2; (Lord et al., 2012) is a semi-structured interview and series of activities to assess behaviors indicative of autism. A Module 4 score consistent with ASD was required for inclusion. The ADOS-2 Module 4 was administered by research-reliable personnel.

*Wechsler Abbreviated Scale of Intelligence* (WASI; (Wechsler, 2011) is a measure of cognitive ability used to obtain a quick, reasonable estimate of an adult’s intellectual functioning (IQ ≥ 70 required). The composite IQ score was used.

*Social Communication Questionnaire* (Rutter et al., 2003) is a screening tool for ASD completed by parents or other adult informants. Scores of 15 or higher are highly suggestive of ASD.

### Intervention

The SENSE Theatre® intervention consisted of ten 2-hour sessions that occurred once a week (i.e., Saturday) as well as two days of technical and dress rehearsals, which culminated in the performance of a 45-minute play.

The SENSE Theatre® approach involves: (1) highly interactive, trained **peers** to facilitate social engagement and communication, (2) theatrical **play** which enhances motivation to engage with others, and (3) a **performance** approach allowing participants to actively practice newly learned skills in a safe, supportive environment. The theatre provides an inclusive space with receptive and trained social partners. Thus, it gives the autistic adult the chance to observe and actively engage in reciprocal social interactions with accepting peers who serve as expert models of social communication. The original SENSE Theatre® copyrighted manual was generally applied with modifications. Specifically, the implementation for the adult participants included: (1) having trained adult peers, (2) more age-appropriate theatrical play themes (e.g., becoming an adult, social relationships), and (3) individualized character development (e.g., creation of personal monologues and performance goals).

All participants completed pretest (before the EXP group treatment), posttest (after EXP treatment), and follow-up assessments (two months after EXP treatment). The EXP group received the SENSE Theatre® intervention first. The WLC group completed the intervention after the final follow-up visit; they were not assessed after the treatment.

Participants were asked to keep non-project treatments stable for at least one month prior and without anticipated changes in the frequency or intensity of these treatments throughout the duration of the study. Participants kept all outside relevant interventions (i.e., social and/or communication skills) stable. Of the total sample, 3 participants initiated psychotherapy specifically for anxiety/depression at the mid-point (WLC = 1, EXP = 1) or posttest (EXP = 1). One other participant in the WLC started an SSRI after post-intervention assessments.

#### Peer Selection

Peers were 18 years of age or older and primarily recruited among Vanderbilt University undergraduate students (e.g., theatre, neuroscience, psychology majors) who sought clinical and research training opportunities. Prospective peers completed an interview with the PI and key personnel trained in clinical psychology to confirm their good reciprocal social communication skills and expressed interest in working with individuals with ASD. The comprehensive interview also ascertained prior personal or professional experience working with autistic or neurodiverse individuals. Involvement in the program was voluntary, yet written consent was required.

Casting and peer assignment was determined by the PI and the Theatre Director based on theatrical experience, understanding of the model, and previous experience working with people with disabilities. When possible, peers and participants were matched based on sex/gender and age; however, other important determinants included the personality and skill set of the peer and functioning level of the participant.

#### Peer Training

The intervention was supervised by the PI and clinical research staff, yet primarily implemented by trained peers. The peers participated in a full day of comprehensive training on ASD, behavioral intervention techniques, and the SENSE Theatre® model. The training involved formal presentations (e.g., autism, core objectives), invited speakers (e.g., adult with ASD, parent of an adult with ASD), relevant topics (e.g., confidentiality), and practice of theatrical strategies (e.g., role-play, mirroring). Videotaped role-plays of target skills simplified for use with the peers (Bellini, 2006; Maurice et al., 1996) were provided. The peer-mediated intervention follows a hierarchical support model that involves active shaping of support in the beginning and gradual fading of supervision as skills are acquired. Weekly 15- to 30-min meetings were conducted with the peers prior to intervention sessions to answer questions, review objectives, and prevent drift. At the end of each day, process sessions gave peers the opportunity to discuss their experiences, obtain suggestions, and receive supportive feedback.

#### Treatment Fidelity & Acceptability

As in previous studies, design, training, and delivery fidelity were measured (Ory et al., 2002). Design fidelity was addressed by utilizing outlined sessions and training logs. Training fidelity was tested by an exam containing 20 questions pertaining to basic knowledge of ASD, the SENSE Theatre® model, and behavioral methods, conducted at the beginning and the end of the full day training. Delivery fidelity was implemented during sessions 1, 3, 5 and 7 by rating peer implementation based on the 10 core objectives and 6 behavioral techniques using a behaviorally anchored five-point Likert scale reported as percentages. Peers who achieved a score of 4 or 5 were deemed satisfactory.

### Dependent Measures

We examined the significance and size of treatment effects on social brain, cognition, behavior and function using objective measures, naïve raters, and standardized clinical outcomes.

#### Measure of Social Brain -– Primary Target

*ERP Incidental Memory for Faces (IFM) Paradigm (*Key & Corbett, 2014*)*, is a non-verbal measure of social salience; specifically, face memory. Stimuli include social and nonsocial stimuli; 51 color photographs of unfamiliar faces (Radboud Faces Database (Langner et al., 2010), 51 color photographs of unfamiliar houses (façade view), and a drawing of a yellow smiley face. One of the unfamiliar faces and one of the houses was randomly selected and repeated 50 times throughout the experiment, yielding a unique set of 50 repeated faces and houses for each participant. Each of the remaining face/house stimuli was presented once (50 trials). From the viewing distance of 90 cm, the stimuli subtended visual angles of 19˚ (h) x 16˚(w). The yellow smiley face was 14.5 cm (9.21˚) in diameter. All stimuli were presented in random order for 1500 ms with a random inter-stimulus interval of 1300–1600 ms to prevent habituation and development of trial onset expectations. To monitor attention to the stimuli, participants were asked to press a response button when they saw the yellow smiley face (10 trials total, brain responses to this stimulus was not analyzed). Stimulus presentations were controlled by E-prime (v.2.0, PST, Inc., Pittsburgh, PA). The task (210 trials) lasted approximately 12 min. Although we are examining a parietal response overlapping the 300-500ms window, it is not the classic P300 because the task is passive, and the repeated and novel stimuli are presented with equal probability (not an oddball). We interpret the observed ERP component to represent change in activity of the fronto-parietal attentional control network (Cole et al., 2014; Lin et al., 2019) as part of the ‘old/new’ response previously labeled ‘P600’ (400-900ms window; (Wilding, 2000) in studies of active memorization and known to occur earlier for face stimuli (255–650 ms; Nelson et al., 1998).

*EEG Acquisition*. Each participant was tested individually and in accordance with best practices for EEG data collection in ASD (Webb et al., 2015). The ERP signals were recorded using 128-channel hydrocel sensor net (Electrical Geodesics, Inc; Eugene, OR) with vertex reference and NetAmps 400 amplifier. The EEG was recorded using NetStation (v. 5.4), sampled at 250 Hz with filters set at 0.1–100 Hz. EEG signal was continuously monitored and during periods of motor activity or inattention, stimulus presentation was suspended until behavior settled. Single-trial ERPs were derived by segmenting EEG on stimulus onset to include a 100-ms baseline and a 900-ms post-stimulus interval and prepared for statistical analyses using standard procedures (e.g. (Key et al., 2013)). Participants with less than 10 artifact-free trials per condition were excluded from analyses. Incidental face memory (IFM) was quantified as the difference in mean ERP amplitudes between repeated and single presentations at the parietal scalp locations within 250–500 ms after stimulus onset (Corbett et al., 2016; Key & Corbett, 2014).

#### Measure of Social Cognition

*Wechsler Memory Scale (WMS-III) Faces* (WMS-F) (Wechsler, 1997). The WMS-F task examines the extent to which adults can recognize and recall a series of 24 photographs of faces. In the task, the participant is initially exposed to 24 faces for 2-sec each, then asked to identify the previously shown faces in a series of 48 faces (Faces I). Following a 30-min delay, the participant is again asked to identify the faces amidst 48 faces (Faces II). WMS-F has been used in studies showing that adults with ASD are impaired in face memory (Williams et al., 2005) and it has also been used to test the impact of face expertise training in ASD (Faja et al., 2012).

#### Measure of Social Behavior

*Contextual Assessment of Social Skills* (CASS**)** (Ratto et al., 2011) is a direct observation measure of social competence explicitly developed for use with individuals with ASD. The CASS consists of two brief role-play conversations with two similar-age, opposite-gender, unfamiliar confederates. It measures the extent to which an individual adapts behavior to changing social context across the Interested condition (I-CASS; confederate shows social interest, engaged verbal and non-verbal demeanor) and Bored condition (B-CASS; another confederate displays bored/disinterested demeanor with minimal eye contact and verbal and nonverbal responding). Consistent with other studies (Corbett et al., 2023), only the CASS-I was used in the analysis as past studies show the CASS-I condition yielded variables sensitive to treatment effects (Dolan et al., 2016; Rabin et al., 2018; White et al., 2015). The CASS was developed for young adults with ASD and has been used to measure treatment outcomes in adults (White et al., 2015) as well as in children and adolescents with ASD (Corbett et al., 2023; Dolan et al., 2016). Based on prior studies reporting demonstrable CASS changes following a social skills intervention (Corbett et al., 2023; Laugeson et al., 2009), two indices were selected: *Vocal Expressiveness* (degree to which the participant varies the tempo, pitch, tone, volume and/or rhythm of speech) and *Quality of Rapport* (degree of rapport and reciprocity between the participant and confederate). Raters were trained to reliability and naïve to group assignment or assessment period when coding video recordings.

#### Measure of Social Functioning

*Social Responsiveness Scale, Second Edition* (SRS-2) (Constantino & Gruber, 2012) covers several areas of behavior characteristic of autism: namely, Awareness, Cognition, Communication, Motivation and Restricted, Repetitive Behaviors. Standard scores of 60 or higher represent clinically significant difficulty in reciprocal social functioning. Due to the age of the participants and inconsistent access to informants, the self-report form was used. Internal consistency ranges from 0.77 to 0.90 and test-retest ranges from 0.77 to 0.88. The SRS has been used as a functional outcome measure in treatment studies in ASD (Corbett et al., 2016; Hardan et al., 2012; Hendren et al., 2016; LaGasse, 2014; Laugeson & Park, 2014; Yui et al., 2012). The SRS has been validated for use in adults, showing concurrent and predictive validity, and strong convergent and discriminant validity (Chan et al., 2017). Also, strong correlations with face memory and the SRS have been shown supporting its use as a linked functional outcome of the treatment (Corbett et al., 2016).

#### Measure of Adaptive Functioning

*Adaptive Behavior Assessment System-Third Edition* (ABAS-3) (Harrison & Oakland, 2015) is a measure of adaptive skills in the home and community. Participants completed the Adult self-report. Test-retest reliability for the self-report is 0.65-0.88 for the adaptive skill areas, 0.82-0.89 for the domains, and 0.87 for the general adaptive composite. Previous SENSE Theatre® research (Corbett et al., 2014b) has shown improvement in adaptive functioning of youth with ASD using the ABAS Parent. The ABAS Adult has been used to measure adaptive skills in adults with ASD (Oswald et al., 2018; Wallace et al., 2016). To examine socially relevant adaptive skills, the Social Composite Subdomain was examined in analyses.

### **Community Involvement**

The study design and intervention modifications were informed by key stakeholders (e.g., autistic adults, parents of individuals on the autism spectrum) that participated in a Community Engagement Studio (CES) held one year prior to the initiation of the study. Information gleaned from the stakeholders guided the length of treatment, selection of dependent measures, and content of the theatrical play themes. Moreover, team members of the theatre-based program are autistic adults.

### **Statistical Analyses**

A series of Analysis of Covariance (ANCOVA) models were used to test the between-group differences on each dependent variable at the immediate posttest and at the follow-up periods separately using the pretest values as a covariate. Independent samples t-tests were used to identify statistically significant differences on all pretest dependent variables. Analyses were performed using SPSS (IBM SPSS Statistics for Macintosh, Version 28.0).

## Results

### Fidelity Results

Delivery fidelity was conducted by research-reliable trainers examining the extent to which peers implemented the program as intended and could range from 0 (no objectives met) to 100 (complete fidelity). Fidelity was conducted during sessions 1, 3, 5, and 7. The mean ratings for the quality of peer implementation of Behavioral Techniques were 95.17 (4.63), 96.89 (5.25), 96.76 (4.37), and 99.00 (2.24), respectively and ratings for the Core Objectives were 97.44 (4.13), 97.21 (3.61), 98.06 (2.41), and 98.20 (4.02), respectively. The results suggest robust and consistent fidelity over time. Training Fidelity was conducted by peers completing pre- and post-testing. The pre-test results for peer training were 0.75 (0.12), while post-test results were 0.85 (0.08) showing good knowledge at baseline and significant improvement following training indicating strong fidelity.

### Preliminary Results

Independent samples t-tests compared initial pretest (T1) difference between the groups on diagnostic and dependent variables. There were no pretest differences for any of the diagnostic measures (age, ADOS or IQ) or dependent variables (See Table 1).

### Primary Results on Dependent Outcome Measures

Multiple levels of Social Competence were measured at Pretest (T1), Posttest (T2) and a two-month Follow-up (T3). ANCOVA assumes homogeneity of variance of the dependent variable is equal across the groups. The homogeneity of variance assumption was tested using Levene’s Test of Equality of Error Variance and it was not violated for any of the dependent variables (all *p* > 0.05). Results for each level of Social Competence are reported below and outlined in Table 2.

**Table 2:** Means, standard deviations, and analysis of covariance statistics at posttest

Variable	EXP		WLC		*F* ratio	*df*	*p*	*d*
	*M*	*SD*	*M*	*SD*				
ERP/IFM	0.6588	0.90058	-0.1604	1.17511	6.315	1,30	**0.016**	0.79
WMS-III (Faces II)	8.35	3.242	7.88	2.740	1.987	1,44	0.166	0.16
CASS								
Vocal Expressiveness	4.82	1.708	4.52	1.648	0.007	1,42	0.932	0.18
Quality of Rapport	5.43	1.409	5.13	1.262	1.539	1,44	0.221	0.22
SRS-2								
Total Score	63.23	9.107	67.50	10.338	3.89	1,43	**0.05**	-0.44
Social Cognition	60.95	7.730	65.58	10.172	3.602	1,43	0.064	-0.51
Social Communication	62.55	9.917	65.79	11.069	4.314	1,43	**0.044**	-0.31
Social Motivation	61.23	9.631	69.46	11.002	12.75	1,43	**< 0.001**	-0.79
Social Awareness	60.50	8.371	58.92	10.008	0.002	1,43	0.965	0.17
SCI	62.73	8.908	67.08	10.198	5.905	1,43	**0.019**	-0.45
ABAS Social Composite	93.30	15.420	83.87	15.855	5.50	1,43	**0.024**	0.60

*Note.* EXP = Experimental Group; WLC = Waitlist Control Group; WASI-II = Wechsler Abbreviated Scale of Intelligence Second Edition; ADOS-2 = Autism Diagnostic Observation Schedule, Second Edition; SCQ-L = Social Communication Questionnaire, Lifetime Form; ERP/IFM = Event Related Potential/Incidental Face Memory; WMS-III (Faces II) = Wechsler Memory Scale, Third Edition—Faces II Subtest; CASS = Contextual Assessment of Social Skills; SRS-2 = Social Responsiveness Scale, Second Edition (SCI Subscale = Social Communication and Interaction); ABAS-3 = Adaptive Behavior Assessment System Third Edition

### Social Brain

**Fig. 2:**
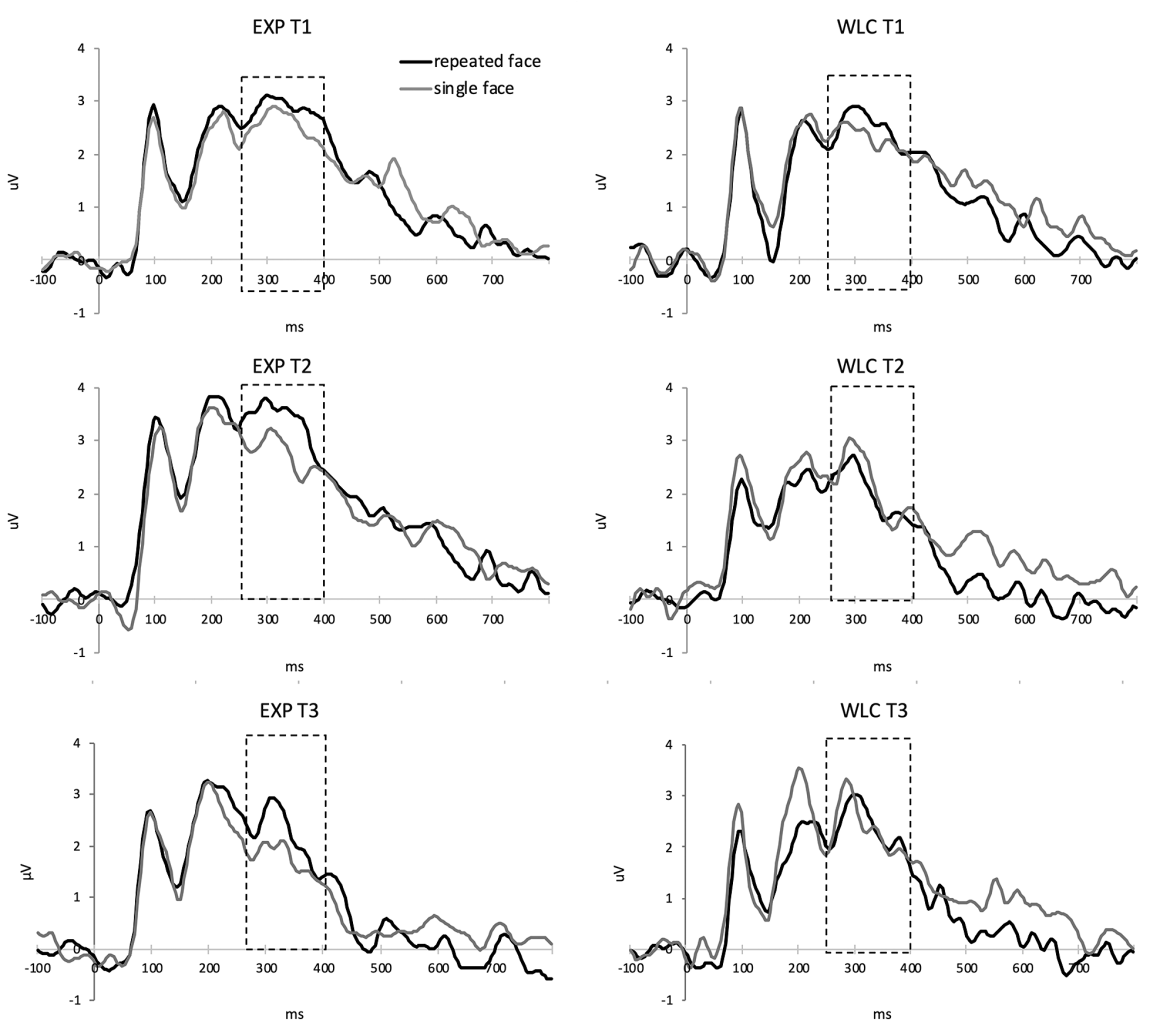
Incidental Face Memory Event-Related Potentials for EXP and WLC groups at T1 (Pretest), T2 (Posttest), and T3 (Follow-up). Note: EXP = Experimental; WLC = Waitlist Control; T1 = Pretest; T2 = Posttest; T3 = Follow-up; ms = milliseconds. Rectangular marker identifies the time window used in the analysis

Using confirmatory analysis based on previous studies, it was predicted that the IFM index (repeated - single face difference score at Pz) would not be significantly different between the groups at baseline (T1; see Table 1) but would be larger in the EXP than WLC group at Posttest (T2). Results demonstrated that the groups did not differ at T1 (See Table 1). At T2, there was a significant group difference, F(1,30) = 6.315, *p* = 0.016, η^2^ = 0.139, d = 0.79, showing higher IFM index in the EXP than WLC group (See Fig. 2; Table 2).

### Social Cognition

It was hypothesized that adults in the EXP group would demonstrate significantly better social cognition via the Wechsler Memory Scale (WMS), Faces II Subtest at posttest compared to the WLC group. However, results demonstrated no significant between-group differences at T2 (Table 2).

### Social Behavior

Based on previous studies in youth (Corbett et al., 2023), it was hypothesized that adults in the EXP group would demonstrate significantly better posttest social behavior on the I-CASS than adults in the WLC. While controlling for pre-test scores, no significant differences between groups were observed at posttest for the I-CASS variables of interest (Vocal Expression, Quality of Rapport) (Table 2).

### Social Functioning

**Fig. 3:**
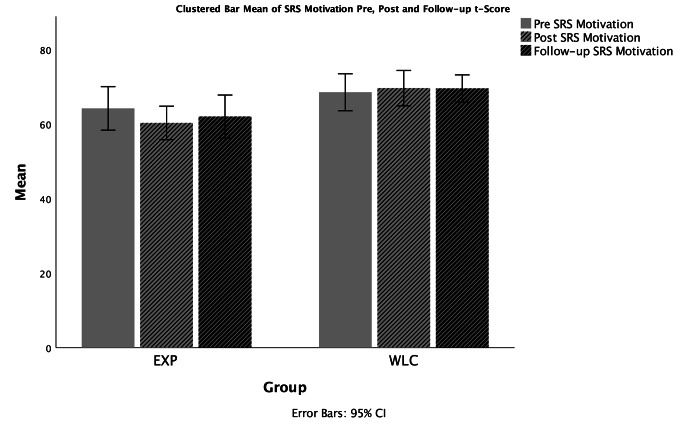
Clustered Bar Mean of Social Responsiveness Scale Motivation for Pretest, Posttest and Follow-up for EXP and WLC. Note: SRS = Social Responsiveness Scale; Pre = Pretest; Post = Posttest; EXP = Experimental; WLC = Waitlist Control; Mean = Mean for Motivation T-Scores.

Adults in the EXP group were expected to demonstrate significantly more growth from pretest to posttest on functional social outcomes (SRS, ABAS). Results largely supported the hypothesis as the EXP group reported significant functional differences on the SRS in several areas of social functioning including Communication (*p* = 0.044), Social Communication and Interaction (SCI) (*p* = 0.019), Social Motivation (p < 0.001; Fig. 3), and the SRS Total Score (*p* = 0.05). There was also a trend for Cognition (*p* = 0.064). Further, participants in the EXP group demonstrated a significant increase in adaptive social functioning compared to the WLC group as measured by the ABAS Social Composite Score (*p* = 0.024). Full SRS and ABAS results are available in Table 2.

### Follow-up (T3)

It was hypothesized that the observed gains in social competence at posttest would be maintained at follow-up. Except for Social Motivation, F(1,40) = 4.445, *p* = 0.041, η^2^ = 0.100, d = -0.77 (Fig. 3), there were no significant gains maintained at follow-up across the social cognition, behavioral and functional outcomes (*p* > 0.05). Complete results are displayed in Table 3.

**Table 3:** Means, standard deviations, and analysis of covariance statistics at follow-up

Variable	EXP		WLC		*F* ratio	*df*	*p*	*d*
	*M*	*SD*	*M*	*SD*				
ERP/IFM	0.469	1.279	-0.77	1.869	1.161	1,37	0.288	0.346
WMS-III (Faces II)	8.35	4.052	8.05	3.184	0.965	1,42	0.331	0.08
CASS								
Vocal Expressiveness	4.30	1.917	4.26	1.485	0.026	1,39	0.873	0.02
Quality of Rapport	5.00	1.168	4.85	1.226	0.438	1,40	0.512	0.13
SRS-2								
Total Score	62.76	10.183	65.77	8.815	0.746	1,40	0.393	-0.32
Social Cognition	62.00	7.376	64.95	9.529	0.898	1,40	0.349	-0.35
Social Communication	62.05	10.181	65.45	11.083	3.217	1,40	0.080	-0.32
Social Motivation	61.48	12.331	69.55	8.262	4.445	1,40	**0.041**	-0.77
Social Awareness	58.10	9.110	56.55	8.187	0.073	1,40	0.789	0.18
SCI	62.43	10.127	66.77	10.071	2.512	1,40	0.121	-0.43
ABAS Social Composite	92.05	13.559	85.33	15.236	2.499	1,39	0.122	0.47

*Note.* EXP = Experimental Group; WLC = Waitlist Control Group; WASI-II = Wechsler Abbreviated Scale of Intelligence Second Edition; ADOS-2 = Autism Diagnostic Observation Schedule, Second Edition; SCQ-L = Social Communication Questionnaire, Lifetime Form; ERP/IFM = Event Related Potential/Incidental Face Memory; WMS-III (Faces II) = Wechsler Memory Scale, Third Edition—Faces II Subtest; CASS = Contextual Assessment of Social Skills; SRS-2 = Social Responsiveness Scale, Second Edition (SCI Subscale = Social Communication and Interaction); ABAS-3 = Adaptive Behavior Assessment System Third Edition

## Discussion

Despite clear evidence that social challenges in ASD persist and may worsen in adulthood (Beadle-Brown et al., 2002; Seltzer et al., 2004), comparatively few social skill interventions (Kandalaft et al., 2013; Koehne et al., 2016; Laugeson et al., 2012; Spain & Blainey, 2015; Taylor et al., 2012; White et al., 2015) or RCTs (e.g., Ashman et al., 2017; Gantman et al., 2012; Oswald et al., 2018) exist. To address this notable gap, the current study used a multilevel social competence framework (neural, cognitive, behavioral, functional) to examine the impact of SENSE Theatre®, an intervention with established efficacy in youth with ASD, in a group of adults with ASD randomized to EXP or WLC conditions.

It was postulated that the key components of peers (e.g., peer-mediation and support), play (e.g., theatrical methods and role play) and performance (e.g., actively engaging in social communication with and in front of others) set the stage to target and enhance core aspects of social competence in autism. Specifically, via increased social attention participants may become better able to recognize individual peers and more adaptively interact with others, which increases their motivation to interact more often, for longer periods, and in more contexts.

The primary hypothesis, that adults with ASD in the EXP group would demonstrate significantly greater IFM posttest than the WLC group, was confirmed and consistent with previous studies (Corbett et al., 2016, 2019, 2023; Ioannou et al., 2020). As explicated below, the finding suggests the intervention enhances salience for relevant social stimuli. While there were no *direct* effects on social cognition (WMS) or behavior (i.e., Vocal Expressiveness, Overall Rapport) at posttest, adults in the EXP group demonstrated significantly more gains on functional (SRS Social Communication, Motivation) and adaptive (ABAS Social) outcomes at posttest which were partially maintained at follow-up.

Face memory is a fundamental social skill necessary for appropriate social development in mammals (Adolphs, 1999) enabling recognition and categorization of conspecifics (Sclafani et al., 2016). Frequently observed deficits in face memory (Key & Corbett, 2014; Langdell, 1978; Osterling et al., 2002; Webb et al., 2010; Weigelt et al., 2012) persist into adulthood (O’Hearn et al., 2014; Williams et al., 2005) and are associated with many aspects of social engagement. Therefore, face memory has been proposed as a primary target of engagement in interventions aimed at enhancing social competence (Corbett et al., 2014a). The increase in social salience of faces reflected in the greater ERP amplitude indexing incidental memory for the repeated image in the current and previous studies of SENSE Theatre® (Corbett et al., 2016, 2019; Ioannou et al., 2020) lends strong support for the notion that the treatment results in greater motivational value placed on social stimuli.

Despite the significant changes in incidental face memory outlined above, there were no group differences on the WMS-III face task (Wechsler, 2009), which has previously revealed impairment in autistic adults (Williams et al., 2005). The WMS-III has been used to show changes in face memory following face expertise training (Faja et al., 2012); however, that intervention employed explicit rule-based skills training in face identification and memory (e.g., configural processing, core features, and shifting attention within stimuli). In contrast, the SENSE Theatre® intervention does not use direct educational approaches, rather it takes a performance-based approach targeting broader social competence skills. Additionally, the WMS-III may be considered a challenging task with multiple immediate (48) and delayed stimuli (48). Anecdotally, several participants remarked on the difficulty and length of the task. Thus, it is unclear if the measure’s level of difficulty or repetition resulted in floor effects rendering it less sensitive as a treatment outcome measure for some types of interventions.

In addition, the experimental treatment did not have a significant effect on the CASS posttest measures of Vocal Expressiveness or Overall Rapport. The results are somewhat similar to a recent RCT of [SENSE Theatre® in youth that did not show immediate gains on these measures at posttest, rather the follow-up behavioral indices were mediated by posttest IFM (Corbett et al., 2023). It may be the case that it takes time for gains in the treatment to translate into social interaction with novel peers; however, follow-up measures were also not significant. While the CASS has been used in youth and adults with ASD, it may not be an optimal measure for repeat testing. At posttest and follow-up, some adults shared that they were anticipating meeting new peers that would show interest or boredom behavior, respectively. Such awareness calls into question the utility of using the CASS as a behavioral outcome for some adults on the spectrum. The task uses natural conversation that may be valuable during an initial exposure, yet the insight of the adult participants suggests it may have limited ecological validity for re-exposure. Turner-Brown and colleagues (2008) used behavioral observation to examine various components of social communication and found no substantial post-intervention improvement. The specific type of social communication measure that may be sensitive to treatment response in adults warrants additional study.

One of the primary goals of a social skills program is to demonstrate perceived functional gains in daily life with other people. In the current study, this was accomplished via self-report in key areas of social and adaptive functioning. For a social skills program to be impactful, improvement needs to be experienced and observed in daily life. Indeed, core diagnostic symptom areas were positively impacted including reciprocal social communication and interaction with others. Autistic adults reported better adaptive functioning in their day-to-day communication and engagement with others following the treatment.

In addition to social communication, the strongest group differences were reported by the EXP group regarding social motivation, which lends support for the idea that SENSE Theatre® contributes to increased social salience for peer engagement. The subscale pulls for interest and comfort in social settings and interactions with other people. It has been suggested that interventions that improve social motivation by increasing the salience of social stimuli should enhance social functioning (Chevallier et al., 2012). Face memory measured by IFM is considered an index of social salience, and remembering a face informs the selection of adaptive social behavior, thereby increasing the likelihood of social success (Corbett et al., 2014a; Hauck, 1998). It is notable that the strongest outcomes were related to aspects of social motivation pertaining to neural and core functional competence. As with previous studies in youth showing strong correlations with face memory and the SRS (Corbett et al., 2016), the current findings suggest that SENSE Theatre® is effective in impacting salience of social information (IFM), which in turn results in improved social functional outcomes (SRS) for adults with ASD.

Intervention research in adults with ASD has underscored the lack of generalizability in clinical efficacy to real-world contexts (Ashman et al., 2017). While many post-treatment gains were not maintained, significant differences in social motivation remained at follow-up. As stated, it has been hypothesized that engaging with supportive peers enhances attention to and interest in social stimuli, and this increase in social salience leads to more generalized social attention and motivation to interact with other people (Corbett et al., 2014a). Collectively, the results highlight the importance of targeting social salience to broadly improve social competence.

### Strengths, Limitations, and Future Directions

There are several strengths of this preliminary RCT, which include careful characterization of the sample, inclusion of multimodal objective measures, naïve raters for behavioral protocols, standardized clinical outcomes, and random assignment. Moreover, in contrast to many studies in ASD, nearly half of the sample was female as the study team actively recruits autistic females for greater representation. Despite these assets, there were limitations in participant representation such that the sample did not include individuals with intellectual disability, and most of the participants were White. There was also larger attrition than expected due, in part, to the COVID-19 pandemic, such that even after restrictions were eased, some participants remained reluctant about meeting in-person and thereby dropped from the study after enrollment. Also, in contrast to studies in youth, in which parental involvement facilitated retention, some adult participants did not have invested family members or friends to provide encouragement to remain in the treatment. Though the selection of peers has been historically based on an interview conducted by the PI and study personnel, the addition of a quantitative measure of social communication skills may help to identify necessary skills to serve as an optimal peer.

Future studies are aimed at addressing the acknowledged limitations to include a more representative sample. While the study team utilized recruitment resources to enroll minority groups, consideration of expanded explanation of potential treatment benefits and compensation may increase interest and commitment. Finally, a careful examination of outcome measures that may be more ecologically valid for adults with ASD is being taken into consideration. It may be that other treatment gains are not captured by the selection of measures, such as receptive and expressive language, emotion regulation, and positive impact on peer relationships in the community. Therefore, greater consideration of functional outcome measures is warranted.

As with many treatment studies, the findings suggest improvement in specific skills; however, the extent to which such skills generalize to other people, settings and contexts is limited. Future work is needed to determine factors that may build on the initial findings to improve generalization of skills. Treatment approaches that generalize to consequential outcomes are needed and an aim of future endeavors.

## Conclusions

Few treatments exist to target social competence in autistic adults. The SENSE Theatre® intervention increases salience of social stimuli in the form of incidental face memory and motivation to engage socially with others. Enhanced social interest provides the opportunity for autistic adults to communicate and engage with others adaptively in their daily lives. Taken together, results lend support to the idea that improving motivation by increasing the salience of social stimuli can lead to improved social functioning (Chevallier et al., 2012). Findings extend previous research supporting the inclusion of trained peers, utilization of innovative theatre techniques and an active performance-based approach allowing autistic adults the opportunity to learn and engage in reciprocal social interactions in a safe, supportive, and inclusive setting. The theatre provides a welcoming environment in which to learn, practice and perform social skills alongside receptive and encouraging social partners. Clinical interventions, such as SENSE Theatre® that go beyond mere instruction of discrete social skills to promote active, dynamic and reinforced performance, increase the likelihood that growth will be maintained and generalized to other people, settings and contexts.

## Footnotes

1 At the time of writing this manuscript there is debate regarding the use of terminology and whether *person-first* language in which the individual (e.g., adult) is referenced before the condition (e.g., autism) or whether *identity-first* language (e.g., autistic adult) should be used. Because such issues have not been resolved, we have opted to take a mixed terminology approach. Similarly, we will use the terms *autism*, *autism spectrum disorder* and *autistic* interchangeably.

## Declarations

### Public Health Significance

Few treatments exist to target social competence in adults with autism spectrum disorder (ASD). The present study provides preliminary evidence for a peer-mediated, theatre-based treatment to enhance salience of social stimuli and generalized effects on social functioning in adults with ASD.

## References

[CR1] Achenbach, T. M. (2001). *Manual for the ASEBA School-Age forms & profiles*. University of Vermont, Research Center for Children, Youth, & Families.

[CR2] Adolphs, R. (1999). Social cognition and the human brain. *Trends in Cognitive Neuroscience*, *3*, 469–479. 10.1016/s1364-6613(99)01399-6 10562726

[CR3] Adolphs, R. (2001). The neurobiology of social cognition. *Current Opinion in Neurobiology*, *11*, 231–239. 11301245 10.1016/s0959-4388(00)00202-6

[CR4] APA. (2013). *Diagnostic and statistical manual of mental disorders, Fifth Edition (DSM-5)*. American Psychiatric Association.

[CR5] Ashman, R., Banksa, K., Philip, R. C. M., Walley, R., & Stanfield, A. C. (2017). A pilot randomised controlled trial of a group based social skills intervention for adults with autism spectrum disorder. *Research in Autism Spectrum Disorders*, *43–44*, 67–75. 10.1016/j.rasd.2017.08.001.

[CR6] Baron-Cohen, S. (1995). *Minblindness: An essay on autism and theory of mind*. MIT Press.

[CR7] Beadle-Brown, J., Murphy, G., Wing, L., Gould, J., Shah, A., & Holmes, N. (2002). Changes in social impairment for people with intellectual disabilities: A follow-up of the Camberwell cohort. *Journal of Autism and Developmental Disorders*, *32*(3), 195–206. 12108621 10.1023/a:1015401814041

[CR8] Bellini, S. (2006). *Building social relationships: A systematic aproach to teaching social interaction skills to children and adolescents with autism spectrum disorders and other social difficulties*. Autism Asperger Publishing Company.

[CR9] Buescher, A. V., Cidav, Z., Knapp, M., & Mandell, D. S. (2014). Costs of autism spectrum disorders in the United Kingdom and the United States. *JAMA Pediatr*, *168*(8), 721–728. 10.1001/jamapediatrics.2014.210. 24911948 10.1001/jamapediatrics.2014.210

[CR10] Chan, W., Smith, L. E., Hong, J., Greenberg, J. S., & Mailick, M. R. (2017). Validating the social responsiveness scale for adults with autism. *Autism Research*, *10*(10), 1663–1671. 10.1002/aur.1813. 28639377 10.1002/aur.1813 PMC5648615

[CR11] Chevallier, C., Kohls, G., Troiani, V., Brodkin, E. S., & Schultz, R. T. (2012). The social motivation theory of autism. *Trends in Cognitive Sciences*, *16*(4), 231–239. 10.1016/j.tics.2012.02.007. 22425667 10.1016/j.tics.2012.02.007 PMC3329932

[CR12] Cole, M. W., Repovs, G., & Anticevic, A. (2014). The frontoparietal control system: A central role in mental health. *The Neuroscientist : A Review Journal Bringing Neurobiology, Neurology and Psychiatry*, *20*(6), 652–664. 10.1177/1073858414525995. 24622818 10.1177/1073858414525995 PMC4162869

[CR13] Constantino, J. N., & Gruber, C. P. (2005). *Social Responsiveness Scale*. Western Psychological Services.

[CR14] Constantino, J. N., & Gruber, C. P. (2012). *The Social Responsiveness Scale Manual, Second Edition (SRS-2)*. Western Psychological Services.

[CR92] Corbett, B. A., Blain, S. D., Ioannou, S., & Balser, M. (2016). Changes in anxiety following a randomized control trial of a theatre-based intervention for youth with autism spectrum disorder. *Autism*. 10.1177/1362361316643623. 10.1177/1362361316643623 PMC5633032 27154909

[CR93] Corbett, B. A., Gunther, J. R., Comins, D., Price, J., Ryan, N., Simon, D., . . . & Rios, T. (2011). Brief report: theatre as therapy for children with autism spectrum disorder. *Journal of autism and developmental disorders, 41*(4), 505–511. 10.1007/s10803-010-1064-1. 10.1007/s10803-010-1064-1 PMC3055998 20640592

[CR94] Corbett, B. A., Ioannou, S., Key, A. P., Coke, C., Muscatello, R., Vandekar, S., & Muse, I. (2019). Treatment Effects in Social Cognition and Behavior following a Theater-based Intervention for Youth with Autism. *Developmental neuropsychology, 44*(7), 481–494. 10.1080/87565641.2019.1676244. 10.1080/87565641.2019.1676244 PMC6818093 31589087

[CR16] Corbett, B. A., Newsom, C., Key, A. P., Qualls, L. R., & Edmiston, E. K. (2014a). Examining the relationship between face processing and social interaction behavior in children with and without autism spectrum disorder. *Journal of Neurodevelopmental Disorders*, *6*. 10.1186/1866-1955-6-35. 10.1186/1866-1955-6-35 PMC4150424 25180050

[CR95] Corbett, B. A., Qualls, L. R., Valencia, B., Fecteau, S. M., & Swain, D. M. (2014b). Peer-mediated theatrical engagement for improving reciprocal social interaction in autism spectrum disorder. *Frontiers in Pediatrics, 2*, 110. 10.3389/fped.2014.00110. 10.3389/fped.2014.00110 PMC4193263 25346926

[CR17] Corbett, B. A., Swain, D. M., Coke, C., Simon, D., Newsom, C., Houchins-Juarez, N., & Song, Y. (2014c). Improvement in Social deficits in Autism Spectrum disorders using a Theatre-Based, peer-mediated intervention. *Autism Research*, *7*, 4–16. 10.1002/aur.1341. 24150989 10.1002/aur.1341 PMC3943749

[CR15] Corbett, B. A., Key, A. P., Qualls, L., Fecteau, S., Newsom, C., Coke, C., & Yoder, P. (2016). Improvement in social competence using a Randomized Trial of a Theatre intervention for children with Autism Spectrum Disorder. *Journal of Autism and Developmental Disorders*, *46*(2), 658–672. 10.1007/s10803-015-2600-9. 26419766 10.1007/s10803-015-2600-9 PMC5633031

[CR18] Corbett, B. A., White, S., Lerner, M., Preacher, K. J., Klemencic, M. E., Simmons, G. L., & Key, A. P. (2023). Peers, play, and performance to build social salience in autistic youth: A multisite randomized clinical trial. *Journal of Consulting and Clinical Psychology*. 10.1037/ccp0000821. 37199977 10.1037/ccp0000821 PMC10330829

[CR19] Dolan, B. K., Van Hecke, A. V., Carson, A. M., Karst, J. S., Stevens, S., Schohl, K. A., & Hummel, E. (2016). Brief report: Assessment of intervention effects on in vivo peer interactions in adolescents with Autism Spectrum disorder (ASD). *Journal of Autism and Developmental Disorders*. 10.1007/s10803-016-2738-0. 26886470 10.1007/s10803-016-2738-0 PMC5291172

[CR20] Dudley, K. M., Klinger, M. R., Meyer, A., Powell, P., & Klinger, L. G. (2018). Understanding service usage and needs for adults with ASD: The importance of living Situation. *Journal of Autism and Developmental Disorders*. 10.1007/s10803-018-3729-0. 30145735 10.1007/s10803-018-3729-0

[CR21] Faja, S., Webb, S. J., Jones, E., Merkle, K., Kamara, D., Bavaro, J., & Dawson, G. (2012). The effects of face expertise training on the behavioral performance and brain activity of adults with high functioning autism spectrum disorders. *Journal of Autism and Developmental Disorders*, *42*(2), 278–293. 10.1007/s10803-011-1243-8. 21484517 10.1007/s10803-011-1243-8 PMC3707515

[CR22] Floris, D. L., Wolfers, T., Zabihi, M., Holz, N. E., Zwiers, M. P., Charman, T., & Auti, E. A. L. E. (2021). Atypical brain asymmetry in Autism-A candidate for clinically meaningful stratification. *Biological Psychiatry-Cognitive Neuroscience and Neuroimaging*, *6*(8), 802–812. 10.1016/j.bpsc.2020.08.008. 33097470 10.1016/j.bpsc.2020.08.008

[CR23] Gantman, A., Kapp, S. K., Orenski, K., & Laugeson, E. A. (2012). Social Skills Training for Young Adults with high-functioning Autism Spectrum disorders: A Randomized Controlled Pilot Study. *Journal of Autism and Developmental Disorders*, *42*(6), 1094–1103. 10.1007/s10803-011-1350-6. 21915740 10.1007/s10803-011-1350-6

[CR24] Gauthier, I., & Nelson, C. A. (2001). The development of face expertise. *Current Opinion in Neurobiology*, *11*(2), 219–224. 11301243 10.1016/s0959-4388(00)00200-2

[CR25] Griffin, J. W., Bauer, R., & Scherf, K. S. (2021). A quantitative meta-analysis of face recognition deficits in autism: 40 years of research. *Psychological Bulletin*, *147*(3), 268–292. 10.1037/bul0000310. 33104376 10.1037/bul0000310 PMC8961473

[CR26] Hardan, A. Y., Fung, L. K., Libove, R. A., Obukhanych, T. V., Nair, S., Herzenberg, L. A., & Tirouvanziam, R. (2012). A randomized controlled pilot trial of oral N-Acetylcysteine in children with autism. *Biological Psychiatry*. 10.1016/j.biopsych.2012.01.014. 22342106 10.1016/j.biopsych.2012.01.014 PMC4914359

[CR28] Harrison, P. L., & Oakland, T. (2000). *Adaptive Behavior Assessment System*. Psychological Corporation.

[CR27] Harrison, P. L., & Oakland, T. (2015). *Adaptive Behavior Assessment System,Third Edition (Manual)*. Western Psychological Services.

[CR29] Hashem, S., Nisar, S., Bhat, A. A., Yadav, S. K., Azeem, M. W., Bagga, P., & Haris, M. (2020). Genetics of structural and functional brain changes in autism spectrum disorder. *Transl Psychiatry*, *10*(1), 229. 10.1038/s41398-020-00921-3. 32661244 10.1038/s41398-020-00921-3 PMC7359361

[CR30] Hauck, M., Fein, D., Maltby, N., Waterhouse, L., & Feinstein, C. (1998). Memory for faces in children with autism. *Neuropsychology*, *4*, 187–198.

[CR31] Hendren, R. L., James, S. J., Widjaja, F., Lawton, B., Rosenblatt, A., & Bent, S. (2016). Randomized, Placebo-Controlled Trial of Methyl B12 for children with autism. *Journal of Child and Adolescent Psychopharmacology*. 10.1089/cap.2015.0159. 26889605 10.1089/cap.2015.0159

[CR32] Howlin, P., & Magiati, I. (2017). Autism spectrum disorder: Outcomes in adulthood. *Current Opinion in Psychiatry*, *30*(2), 69–76. 10.1097/YCO.0000000000000308. 28067726 10.1097/YCO.0000000000000308

[CR33] Hyde, K. L., Samson, F., Evans, A. C., & Mottron, L. (2010). Neuroanatomical differences in brain areas implicated in perceptual and other core features of autism revealed by cortical thickness analysis and voxel-based morphometry. *Human Brain Mapping*, *31*(4), 556–566. 10.1002/hbm.20887. 19790171 10.1002/hbm.20887 PMC6870833

[CR34] Ioannou, S., Key, A. P., Muscatello, R. A., Klemencic, M., & Corbett, B. A. (2020). Peer actors and theater techniques play pivotal roles in improving social play and anxiety for children with autism. *Front Psychol*, *11*(Article 908). 10.3389/fpsyg.2020.00908 PMC7269006 32536887

[CR35] Johnson, M. H. (2017). Autism as an adaptive common variant pathway for human brain development. *Developmental Cognitive Neuroscience*, *25*, 5–11. 10.1016/j.dcn.2017.02.004. 28233663 10.1016/j.dcn.2017.02.004 PMC6987822

[CR36] Kandalaft, M. R., Didehbani, N., Krawczyk, D. C., Allen, T. T., & Chapman, S. B. (2013). Virtual reality social cognition training for young adults with high-functioning autism. *Journal of Autism and Developmental Disorders*, *43*(1), 34–44. 10.1007/s10803-012-1544-6. 22570145 10.1007/s10803-012-1544-6 PMC3536992

[CR37] Kennedy, D. P., & Adolphs, R. (2012). The social brain in psychiatric and neurological disorders. *Trends in Cognitive Sciences*, *16*(11), 559–572. 10.1016/j.tics.2012.09.006. 23047070 10.1016/j.tics.2012.09.006 PMC3606817

[CR38] Key, A. P., & Corbett, B. A. (2014). ERP responses to face repetition during passive viewing: A nonverbal measure of social motivation in children with autism and typical development. *Dev Neuropsychol*, *39*(6), 474–495. 10.1080/87565641.2014.940620. 25144259 10.1080/87565641.2014.940620 PMC4142544

[CR39] Key, A. P., Jones, D., & Dykens, E. M. (2013). Social and emotional processing in Prader-Willi syndrome: Genetic subtype differences. *J Neurodev Disord*, *5*(1), 7. 10.1186/1866-1955-5-7. 23536992 10.1186/1866-1955-5-7 PMC3637538

[CR40] Koehne, S., Behrends, A., Fairhurst, M. T., & Dziobek, I. (2016). Fostering Social Cognition through an imitation- and synchronization-based Dance/Movement intervention in adults with Autism Spectrum disorder: A controlled proof-of-Concept Study. *Psychotherapy and Psychosomatics*, *85*(1), 27–35. 10.1159/000441111. 26609704 10.1159/000441111

[CR41] Korkman, M., Kirk, U., & Kemp, S. (2007). *NEPSY 2nd Edition*. Harcourt Assessment.

[CR42] LaGasse, A. B. (2014). Effects of a music therapy group intervention on enhancing social skills in children with autism. *Journal of Music Therapy*, *51*(3), 250–275. 10.1093/jmt/thu012. 25053766 10.1093/jmt/thu012

[CR43] Langdell, T. (1978). Recognition of faces: An approach to the study of autism. *Journal of Child Psychology and Psychiatry*, *19*(3), 255–268. 681468 10.1111/j.1469-7610.1978.tb00468.x

[CR44] Langner, O., Dotsch, R., Bijlstra, G., Wigboldus, D., Hawk, S., & van Knippenberg, A. (2010). Presentation and validation of the Radboud faces database. *Cognition and Emotion*, *24*(8), 1377–1388.

[CR47] Laugeson, E. A., & Park, M. N. (2014). Using a CBT approach to teach social skills to adolescents with autism spectrum disorder and other social challenges: The PEERS® method. *Journal of Rational-Emotive & Cognitive-Behavior Therapy*, *32*(1), 84–97.

[CR46] Laugeson, E. A., Frankel, F., Mogil, C., & Dillon, A. R. (2009). Parent-assisted social skills training to improve friendships in teens with autism spectrum disorders. *Journal of Autism and Developmental Disorders*, *39*(4), 596–606. 10.1007/s10803-008-0664-5. 19015968 10.1007/s10803-008-0664-5

[CR45] Laugeson, E. A., Frankel, F., Gantman, A., Dillon, A. R., & Mogil, C. (2012). Evidence-based social skills training for adolescents with autism spectrum disorders: The UCLA PEERS program. *Journal of Autism and Developmental Disorders*, *42*(6), 1025–1036. 10.1007/s10803-011-1339-1. 21858588 10.1007/s10803-011-1339-1

[CR48] Leigh, J. P., Grosse, S. D., Cassady, D., Melnikow, J., & Hertz-Picciotto, I. (2016). Spending by California’s Department of Developmental Services for persons with autism across demographic and expenditure categories. *PLoS One*, *11*(3), e0151970. 10.1371/journal.pone.0151970. 27015098 10.1371/journal.pone.0151970 PMC4807877

[CR49] Lever, A. G., & Geurts, H. M. (2018). Is older Age Associated with higher self- and other-rated ASD characteristics? *Journal of Autism and Developmental Disorders*, *48*(6), 2038–2051. 10.1007/s10803-017-3444-2. 29349671 10.1007/s10803-017-3444-2 PMC5948271

[CR50] Lin, H. Y., Perry, A., Cocchi, L., Roberts, J. A., Tseng, W. I., Breakspear, M., & Gau, S. S. (2019). Development of frontoparietal connectivity predicts longitudinal symptom changes in young people with autism spectrum disorder. *Transl Psychiatry*, *9*(1), 86. 10.1038/s41398-019-0418-5. 30755585 10.1038/s41398-019-0418-5 PMC6372645

[CR51] Lord, C., Rutter, M., DiLavore, P. C., Risi, S., Gotham, K., & Bishop, S. L. (2012). *Autism Diagnostic Observation schedule (ADOS-2)* (2nd ed.). Western Psychological Services.

[CR52] Lynn, A. C., Padmanabhan, A., Simmonds, D., Foran, W., Hallquist, M. N., Luna, B., & O’Hearn, K. (2018). Functional connectivity differences in autism during face and car recognition: Underconnectivity and atypical age-related changes. *Developmental Science*, *21*(1), 10.1111/desc.12508. 10.1111/desc.12508 PMC5392438 27748031

[CR53] Maenner, M. J., Warren, Z., Williams, A. R., Amoakohene, E., Bakian, A. V., Bilder, D. A., & Shaw, K. A. (2023). Prevalence and characteristics of Autism Spectrum Disorder among children aged 8 years - Autism and Developmental Disabilities Monitoring Network, 11 sites, United States, 2020. *MMWR Surveill Summ*, *72*(2), 1–14. 10.15585/mmwr.ss7202a1. 36952288 10.15585/mmwr.ss7202a1 PMC10042614

[CR54] Marcus, D. J., & Nelson, C. A. (2001). Neural bases and development of face recognition in autism. *Cns Spectrums*, *6*(1), 36–59. 17008831 10.1017/s1092852900022872

[CR55] Maurice, C., Green, G., & Luce, S. C. (1996). *Behavioral intervention for young children with autism*. Pro-ed.

[CR56] McVey, A. J., Dolan, B. K., Willar, K. S., Pleiss, S., Karst, J. S., Casnar, C. L., & Van Hecke, A. V. (2016). A replication and extension of the PEERS(R) for young adults social skills intervention: Examining effects on Social Skills and Social Anxiety in young adults with Autism Spectrum Disorder. *Journal of Autism and Developmental Disorders*, *46*(12), 3739–3754. 10.1007/s10803-016-2911-5. 27628940 10.1007/s10803-016-2911-5 PMC5310211

[CR57] Myers, E., Davis, B. E., Stobbe, G., & Bjornson, K. (2015). Community and Social Participation among Individuals with Autism Spectrum Disorder transitioning to Adulthood. *Journal of Autism and Developmental Disorders*, *45*(8), 2373–2381. 10.1007/s10803-015-2403-z. 25725812 10.1007/s10803-015-2403-z

[CR58] Nelson, C. A. (2001). The development and neural bases of face recognition. *Infant Child Development*, *10*(1–2), 3–18.

[CR59] Nelson, C. A., Thomas, K. M., de Haan, M., & Wewerka, S. S. (1998). Delayed recognition memory in infants and adults as revealed by event-related potentials. *International Journal of Psychophysiology*, *29*(2), 145–165. 9664226 10.1016/s0167-8760(98)00014-2

[CR60] O’Hearn, K., Tanaka, J., Lynn, A., Fedor, J., Minshew, N., & Luna, B. (2014). Developmental plateau in visual object processing from adolescence to adulthood in autism. *Brain and Cognition*, *90*, 124–134. 10.1016/j.bandc.2014.06.004. 25019999 10.1016/j.bandc.2014.06.004 PMC4944205

[CR61] Ory, M. G., Jordan, P. J., & Bazzarre, T. (2002). The Behavior Change Consortium: Setting the stage for a new century of health behavior-change research. *Health Education Research*, *17*(5), 500–511. 12408195 10.1093/her/17.5.500

[CR62] Osterling, J. A., Dawson, G., & Munson, J. A. (2002). Early recognition of 1-year-old infants with autism spectrum disorder versus mental retardation. *Development and Psychopathology*, *14*(2), 239–251. 12030690 10.1017/s0954579402002031

[CR63] Oswald, T. M., Winder-Patel, B., Ruder, S., Xing, G., Stahmer, A., & Solomon, M. (2018). A pilot randomized controlled trial of the ACCESS Program: A Group intervention to improve Social, adaptive functioning, stress coping, and self-determination outcomes in young adults with Autism Spectrum Disorder. *Journal of Autism and Developmental Disorders*, *48*(5), 1742–1760. 10.1007/s10803-017-3421-9. 29234931 10.1007/s10803-017-3421-9 PMC5889958

[CR64] Pallathra, A. A., Calkins, M. E., Parish-Morris, J., Maddox, B. B., Perez, L. S., Miller, J., & Brodkin, E. S. (2018). Defining behavioral components of social functioning in adults with autism spectrum disorder as targets for treatment. *Autism Research*, *11*(3), 488–502. 10.1002/aur.1910. 29341497 10.1002/aur.1910 PMC5890924

[CR96] Rabin, S. J., Israel‐Yaacov, S., Laugeson, E. A., Mor‐Snir, I., & Golan, O. (2018). A randomized controlled trial evaluating the Hebrew adaptation of the PEERS® intervention: Behavioral and questionnaire‐based outcomes. *Autism Research, 11*(8), 1187–1200 10.1002/aur.1974 30095232

[CR65] Ratto, A. B., Turner-Brown, L., Rupp, B. M., Mesibov, G. B., & Penn, D. L. (2011). Development of the Contextual Assessment of Social Skills (CASS): A role play measure of social skill for individuals with high-functioning autism. *Journal of Autism and Developmental Disorders*, *41*(9), 1277–1286. 10.1007/s10803-010-1147-z. 21287253 10.1007/s10803-010-1147-z

[CR66] Rutter, M., Bailey, A., & Lord, C. (2003). *The Social Communication Questionnaire*. Western Psychological Services.

[CR67] Sclafani, V., Rosso, D., Seil, L. A., Calonder, S. K., Madrid, L. A., Bone, J. E., & Parker, K. J., K. J (2016). Early predictors of impaired Social Functioning in Male Rhesus macaques (Macaca mulatta). *PLoS One*, *11*(10), e0165401. 10.1371/journal.pone.0165401. 27788195 10.1371/journal.pone.0165401 PMC5082922

[CR68] Seltzer, M. M., Shattuck, P., Abbeduto, L., & Greenberg, J. S. (2004). Trajectory of development in adolescents and adults with autism. *Mental Retardation and Developmental Disabilities Research Reviews*, *10*(4), 234–247. 10.1002/mrdd.20038. 15666341 10.1002/mrdd.20038

[CR69] Shafritz, K. M., Dichter, G. S., Baranek, G. T., & Belger, A. (2008). The neural circuitry mediating shifts in behavioral response and cognitive set in autism. *Biological Psychiatry*, *63*(10), 974–980. 10.1016/j.biopsych.2007.06.028. 17916328 10.1016/j.biopsych.2007.06.028 PMC2599927

[CR71] Shattuck, P. T., Wagner, M., Narendorf, S., Sterzing, P., & Hensley, M. (2011). Post-high School Service Use among Young adults with an Autism Spectrum Disorder. *Archives of Pediatrics & Adolescent Medicine*, *165*(2), 141–146. 10.1001/archpediatrics.2010.279. 21300654 10.1001/archpediatrics.2010.279 PMC3097532

[CR70] Shattuck, P. T., Steinberg, J., Yu, J., Wei, X., Cooper, B. P., Newman, L., & Roux, A. M. (2014). Disability Identification and Self-Efficacy among College Students on the Autism Spectrum. *Autism Res Treat*, *2014*, 924182. 10.1155/2014/924182. 10.1155/2014/924182 PMC3953486 24707401

[CR72] Spain, D., & Blainey, S. H. (2015). Group social skills interventions for adults with high-functioning autism spectrum disorders: A systematic review. *Autism*, *19*(7), 874–886. 10.1177/1362361315587659. 26045543 10.1177/1362361315587659

[CR75] Taylor, J. L., & Henninger, N. A. (2015). Frequency and correlates of Service Access among Youth with Autism transitioning to Adulthood. *Journal of Autism and Developmental Disorders*, *45*(1), 179–191. 10.1007/s10803-014-2203-x. 25081594 10.1007/s10803-014-2203-x PMC4288981

[CR76] Taylor, J. L., & Seltzer, M. M. (2010). Changes in the autism behavioral phenotype during the transition to adulthood. *Journal of Autism and Developmental Disorders*, *40*(12), 1431–1446. 10.1007/s10803-010-1005-z. 20361245 10.1007/s10803-010-1005-z PMC2910794

[CR74] Taylor, J. L., Dove, D., Veenstra-Vander Weele, J., Sathe, N. A., McPheeters, M. L., Jerome, R. N., et al. (2012). *Interventions for adolescents and young adults with autism spectrum disorders Agency*. for Healthcare Research and Quality (US). 23035276

[CR73] Taylor, J. L., Adams, R. E., & Bishop, S. L. (2017). Social participation and its relation to internalizing symptoms among youth with autism spectrum disorder as they transition from high school. *Autism Research*, *10*(4), 663–672. 10.1002/aur.1709. 27739234 10.1002/aur.1709 PMC5392176

[CR77] Turcotte, P., Mathew, M., Shea, L. L., Brusilovskiy, E., & Nonnemacher, S. L. (2016). Service needs across the Lifespan for individuals with autism. *Journal of Autism and Developmental Disorders*, *46*(7), 2480–2489. 10.1007/s10803-016-2787-4. 27084080 10.1007/s10803-016-2787-4

[CR78] Turner-Brown, L. M., Perry, T. D., Dichter, G. S., Bodfish, J. W., & Penn, D. L. (2008). Brief report: Feasibility of social cognition and interaction training for adults with high functioning autism. *Journal of Autism and Developmental Disorders*, *38*(9), 1777–1784. 10.1007/s10803-008-0545-y. 18246419 10.1007/s10803-008-0545-y PMC2646378

[CR79] Umagami, K., Remington, A., Lloyd-Evans, B., Davies, J., & Crane, L. (2022). Loneliness in autistic adults: A systematic review. *Autism*, *26*(8), 2117–2135. 10.1177/13623613221077721. 35257592 10.1177/13623613221077721 PMC9597154

[CR80] Wallace, G. L., Kenworthy, L., Pugliese, C. E., Popal, H. S., White, E. I., Brodsky, E., & Martin, A. (2016). Real-World Executive functions in adults with Autism Spectrum Disorder: Profiles of Impairment and associations with adaptive functioning and co-morbid anxiety and depression. *Journal of Autism and Developmental Disorders*, *46*(3), 1071–1083. 10.1007/s10803-015-2655-7. 26572659 10.1007/s10803-015-2655-7 PMC5111802

[CR82] Webb, S. J., Jones, E. J., Merkle, K., Namkung, J., Toth, K., Greenson, J., & Dawson, G. (2010). Toddlers with elevated autism symptoms show slowed habituation to faces. *Child Neuropsychology*, *16*(3), 255–278. 10.1080/09297041003601454. 20301009 10.1080/09297041003601454 PMC2989718

[CR81] Webb, S. J., Bernier, R., Henderson, H. A., Johnson, M. H., Jones, E. J., Lerner, M. D., & Westerfield, M. (2015). Guidelines and best practices for electrophysiological data collection, analysis and reporting in autism. *Journal of Autism and Developmental Disorders*, *45*(2), 425–443. 10.1007/s10803-013-1916-6. 23975145 10.1007/s10803-013-1916-6 PMC4141903

[CR83] Wechsler, D. (1997). *WMS-III administration and scoring manual*. The Psychological Corporation.

[CR84] Wechsler, D. (2009). *Wechsler Memory Scale 4th Edition Technical and Interpretive Manual*. Pearson.

[CR85] Wechsler, D. (2011). *Wechsler Abbreviated Scale of Intelligence II* (Vol. Second Edition). PsychCorp.

[CR86] Weigelt, S., Koldewyn, K., & Kanwisher, N. (2012). Face identity recognition in autism spectrum disorders: A review of behavioral studies. *Neuroscience and Biobehavioral Reviews*, *36*(3), 1060–1084. 10.1016/j.neubiorev.2011.12.008. 22212588 10.1016/j.neubiorev.2011.12.008

[CR87] White, S. W., Scarpa, A., Conner, C. M., Maddox, B. B., & Bonete, S. (2015). Evaluating change in Social skills in high-functioning adults with Autism Spectrum Disorder using a laboratory-based observational measure. *Focus on Autism and Other Developmental Disabilities*, *30*(1), 3–12. 10.1177/1088357614539836.

[CR88] Wilding, E. L. (2000). In what way does the parietal ERP old/new effect index recollection? *International Journal of Psychophysiology*, *35*(1), 81–87. https://www.ncbi.nlm.nih.gov/pubmed/10683669. 10683669 10.1016/s0167-8760(99)00095-1

[CR89] Williams, D. L., Goldstein, G., & Minshew, N. J. (2005). Impaired memory for faces and social scenes in autism: Clinical implications of memory dysfunction. *Archives of Clinical Neuropsychology : The Official Journal of the National Academy of Neuropsychologists*, *20*(1), 1–15. 10.1016/j.acn.2002.08.001. 15620811 10.1016/j.acn.2002.08.001

[CR90] Wong, C., Odom, S. L., Hume, K. A., Cox, A. W., Fettig, A., Kucharczyk, S., & Schultz, T. R. (2015). Evidence-based practices for Children, Youth, and young adults with Autism Spectrum disorder: A Comprehensive Review. *Journal of Autism and Developmental Disorders*, *45*(7), 1951–1966. 10.1007/s10803-014-2351-z. 25578338 10.1007/s10803-014-2351-z

[CR91] Yui, K., Koshiba, M., Nakamura, S., & Kobayashi, Y. (2012). Effects of large doses of arachidonic acid added to docosahexaenoic acid on social impairment in individuals with autism spectrum disorders: A double-blind, placebo-controlled, randomized trial. *Journal of Clinical Psychopharmacology*, *32*(2), 200–206. 10.1097/JCP.0b013e3182485791. 22370992 10.1097/JCP.0b013e3182485791

